# Diversity and evolutionary analysis of viruses carried by mosquitoes in Shandong, China

**DOI:** 10.1128/spectrum.01018-25

**Published:** 2025-08-11

**Authors:** Yujie Liu, Xiaohua Zhao, Xiuwei Feng, Wenbing Zhu, Shuo Feng, Meixi Ren, Yingxin Tu, Guoyu Niu, Yujing Zhu

**Affiliations:** 1 Central Sterile Supply Department, Affiliated Hospital of Shandong Second Medical University 576398 , Weifang, China; 2 Shandong Second Medical University 372527 , Weifang, China; 3 Suqian First Hospital 710134 , Suqian, China; Ascension St John Hospital, Detroit, Michigan, USA

**Keywords:** mosquito-associated viruses, *Culex quinquefasciatus*, novel viruses, phylogenetic analysis

## Abstract

Arboviruses represent a growing threat to global public health, as climate change is exacerbating the spread of these vectors and thereby increasing the transmission potential of arboviruses. As primary vectors, mosquitoes harbor remarkably diverse virus populations, broadly classified into mosquito-borne viruses and mosquito-specific viruses. Although metagenomic sequencing has revolutionized viral discovery, geographical variations in mosquito viromes remain poorly characterized. In this study, we conducted comprehensive viral surveillance on 5,051 mosquitoes representing six medically important species (grouped into 14 taxonomic units) collected from Shandong, China, in 2021. Using next-generation sequencing, we generated 3.7 billion clean reads and identified 10 distinct virus species spanning eight virus families. Phylogenetic analyses revealed substantial genomic diversity and complex evolutionary relationships, including two completely novel virus species and several incompletely characterized known viruses. Notably, *Culex quinquefasciatus* mosquitoes exhibited significantly greater viral diversity than other species, although we observed considerable overlap in viral communities across mosquito taxa, suggesting non-host-specific viral maintenance. The presence of two novel viruses in field populations was confirmed by RT-qPCR screening, wherein minimum infection rates of 0.16% and 0.38% were established. Our findings substantially expand the known diversity of mosquito-associated viruses in East Asia and provide critical baseline data for arbovirus surveillance programs. This work highlights the value of systematic virome characterization for predicting emerging arboviral threats and understanding virus-mosquito ecology in changing environments.

**IMPORTANCE:**

Ten viruses, including two novel ones, were found in a study performed on mosquitoes in Shandong, China. It shows viral diversity and coexistence in different species, highlighting host impact on viral communities. The new viruses are prevalent locally, with infection rates of 0.38% and 0.16%. This work advances viral ecology understanding and has public health significance. This study sheds light on the circulation of the identified viruses in Shandong.

## INTRODUCTION

Arboviruses, a collective term for a diverse group of viruses, are transmitted to susceptible vertebrate hosts through the bites of arthropod vectors. Sometimes resulting in various diseases (1, 2). Among the arthropod vectors capable of harboring and transmitting these viruses, mosquitoes and ticks are the most prominent, sometimes resulting in various diseases, particularly in the context of human diseases, as they are responsible for transmitting a wide range of pathogens that cause significant morbidity and mortality globally (3, 4). To date, over 600 arbovirus species have been identified globally, with mosquito-borne arboviruses comprising more than 300 species, comprising a majority of the total arbovirus diversity (5). Furthermore, the spectrum of diseases transmitted by different mosquito species exhibits significant variation. For instance, *Culex* mosquitoes are the primary vectors for epidemic Japanese encephalitis, West Nile fever, and Bancroftian filariasis; *Aedes aegypti* mosquitoes are mainly responsible for the transmission of dengue, chikungunya, yellow fever, Zika virus disease, and Rift Valley fever; and *Anopheles* mosquitoes are the principal vectors for malaria and Malayan filariasis. However, it is important to note that the transmission of these pathogens does not always result in disease manifestation, as the outcome depends on various factors, including host immunity and environmental conditions (6–8).

Shandong Province, ranking as the second most populous province in China, is situated along the eastern coast of the country and the lower reaches of the Yellow River. The province is characterized by a diverse landscape, featuring mountainous and hilly terrain interspersed with interconnected plains and basins. Weifang (118°10′~120°01′N, 35°41′~37°26′E), situated in the western region of the Shandong Peninsula, lies at the heart of the Peninsula City Cluster and shares its northern border with the Laizhou Bay of the Bohai Sea. The climate of Weifang is classified as a warm temperate monsoon-influenced semi-humid continental climate, characterized by concentrated precipitation. The average annual temperature ranges from 11°C to 14°C. During the summer months, temperatures often exceed 25°C, and these warmer conditions, combined with high humidity and frequent rainfall, create optimal environments for mosquito breeding and reproduction. These climatic factors facilitate the transmission of mosquito-borne viruses, making the summer season particularly conducive to increased mosquito vector activity (9). The predominant mosquito species found in Shandong Province comprise *Armigeres subalbatus*, *Culex tritaeniorhynchus*, *Anopheles sinensis*, and *Culex pipiens pallens* (10, 11). Shandong Province, home to numerous ports, has witnessed a significant increase in the frequency of international trade, economic activities, and tourism. The convenience of modern transportation has also led to heightened exposure to a growing number of emerging, reemerging, and imported arboviral threats. Consequently, it is imperative to perform periodic surveys on mosquito populations and the mosquito-borne viruses they harbor, monitor the infection rates and genotypic changes of these viruses, and establish a scientific foundation for the prevention and control of mosquito-borne diseases. In light of these considerations, we elected to undertake mosquito collection and monitoring in Weifang, Shandong Province. Employing macrogenomic analysis, we identified 10 viruses belonging to eight families and conducted phylogenetic analyses. Our research not only contributes to the understanding of mosquito-borne virus diversity but also offers fundamental data on the epidemiological features of these viruses in the region.

## MATERIALS AND METHODS

### Sample collection and processing

For this study, the livestock farming area in Weifang, Shandong Province, was chosen as the study site, with a total of eight permanent monitoring stations established in the vicinity of various farm types, including pig farms, cattle pens, and sheep houses. The monitoring period was selected to coincide with the annual peak in temperature and precipitation in the region, spanning from sunset to early morning of the following day (18:00–06:00). Field collection was conducted using UV mosquito traps (Kongfuxiaoshuai, China). A total of 5,051 mosquitoes were collected during the study period. The collected samples were transferred to the cold chain and stored in the laboratory at −20°C. Initial identification of the samples was performed based on morphological characteristics. Subsequently, the mosquitoes were categorized according to their collection location, and each group of 50 mosquitoes was numbered and stored to establish a detailed file of sample information. To verify the accuracy of species identification, all morphologically identified samples underwent molecular biological validation using the *COI* gene sequence analysis method for species confirmation. To verify the accuracy of species identification, all morphologically identified samples underwent molecular biological validation, employing the *COI* gene sequence analysis method for species confirmation (12).

### Virus RNA extraction

A volume of 1,000 µL of DMEM medium was dispensed into each of the pre-labeled grinding tubes (TissueLyser LT Adapter, QIAGEN), maintained at a low temperature. Subsequently, the samples were homogenized using a tissue grinder (Tissuelyser, Germany) operating at a fixed oscillation frequency of 30 Hz for a duration of 5–10 min until homogeneous samples were achieved. The homogenized samples were centrifuged at 15,000 *× g* for 30 min at a temperature of 4°C. The resulting supernatant was harvested and underwent nucleic acid purification using a viral RNA extraction kit (Tiangen, China). The purified RNA samples can be utilized immediately for subsequent assays or stored at −80°C in an ultra-low temperature freezer for long-term preservation.

### Library construction and sequencing

In total, 14 mixed sample libraries were generated by aspirating 5 µL of RNA from each milled tube and subsequently pooling the aliquots for thorough mixing. This process ensured that each library represented a diverse collection of samples, facilitating comprehensive analysis. Subsequently, pre-treatment was initiated by quantifying the RNA concentration. For samples exhibiting a concentration exceeding 10 ng/µL, the ribosomal RNA removal kit (FastSelect, Vazyme, China) was employed to deplete *rRNA*. Samples with low concentrations (<10 ng/µL) were directly subjected to the subsequent processing step. Sequencing libraries were prepared in accordance with the protocol outlined in the RNA Sequencing Library Preparation Kit (VAHTS Universal V8, Vazyme). The concentration of the library was first measured using a Qubit fluorometer. Subsequently, the fragment distribution of the diluted libraries was assessed using the Agilent 2100 DNA 1000 Kit. Ultimately, the prepared libraries were transferred to Beijing Jiyinga Medical Laboratory Co., Ltd. for quality assessment using a bioanalyzer (Agilent 2100 Bioanalyzer) and real-time fluorescence quantitative PCR. From the quality-assured libraries, DNA nanospheres were generated, loaded onto sequencing chips, and subjected to paired-end sequencing (PE150) using a high-throughput sequencing platform (DNBSEQ-T7, BGI, China).

### Sequence alignment and virus sequence discovery

Initially, the sequencing data were imported into CLC Genomics Workbench v11.0.1 for rigorous quality control. Low-quality reads and adapter sequences were removed, with a quality threshold of Q30 and a minimum length of 50 nucleotides. Next, *rRNA* and genomic sequences originating from the host were efficiently removed using STAR software v2.7.3a. The remaining non-host reads were *de novo* assembled using MEGAHIT software v1.2.9 to generate high-quality contigs. The assembled contigs were then accurately compared with the viral protein database and the non-redundant nucleotide database using *blastx* and *blastn* tools, with an E-value cutoff of <1e-5 to identify potential viral overlapping clusters. The database data were downloaded in August 2024. Finally, sequence clusters with more than 50 read counts and less than 60% sequence identity to known viruses were considered potential novel viruses, warranting in-depth analysis and rigorous validation.

### Virus classification and annotation

Virus classification was performed according to the most recent virus classification report released by the International Committee on Classification of Viruses (ICTV). For each virus family, species demarcation thresholds based on genomic and protein sequence identity were applied as recommended by the ICTV. Sequences with less than 80% nucleotide similarity or less than 90% RNA-dependent RNA polymerase (RdRp) amino acid similarity were identified as novel virus species (13). The nomenclature of viruses primarily follows the principle of territoriality, in conjunction with their general viral taxonomic characteristics. Viruses that have already been assigned to an existing taxonomy are labeled "21W-GJD" to differentiate them from previously described strains.

We analyzed the virus sequences identified in this study for potential open reading frames (ORFs) with a minimum length of 100 amino acids (aa) using ORF Finder (https://www.ncbi.nlm.nih.gov/orffinder/). These ORFs were further compared with related virus reference sequences. To identify the conserved structural domains in each ORF, we searched and analyzed them using the Conserved Domain Search Service (https://www.ncbi.nlm.nih.gov/Structure/cdd/wrpsb.cgi). The potential functions of the conserved domains were predicted through homology with other known viral proteins.

### Phylogenetic analyses

Multiple sequence alignment was carried out using MEGA software (version 10.2.6) employing the ClustalW algorithm with default parameters. Phylogenetic analyses were performed using two distinct methods, selected based on the characteristics of the sequences. For previously reported sequences, phylogenetic trees were constructed using the neighbor-joining (NJ) method with the Kimura-2-parameter model and 1,000 bootstrap replicates to evaluate node reliability. For novel virus sequences identified in this study, maximum likelihood (ML) trees were constructed using IQ-TREE (v2.0), employing the best-fit substitution model selected by ModelFinder based on the Bayesian Information Criterion. Branch support values were calculated using 1,000 ultrafast bootstrap replicates. The resulting phylogenetic trees were visualized and aesthetically refined using FigTree v1.4.4 (http://tree.bio.ed.ac.uk/software/figtree/).

### Detection of the RNA of Shandong Ifla-like virus 1 and 2 in mosquitoes

Quantitative reverse-transcription PCR (RT-qPCR) was employed to detect the presence of the virus in mosquito pools. In this study, the one-step RT-PCR method was utilized, and virus-specific primer-probe sets were described as shown in Table 1. The reaction was carried out in a volume of 25 µL, containing 5 µL 5 × buffer, 1 µL dNTP, 1 µL enzyme mix, 11.75 µL RNase-free water, 0.5 µL each of upstream and downstream primers, 0.25 µL probe, and 5 µL RNA. The qRT-PCR was performed at 50°C for 30 min and 95°C for 15 min, followed by 35 cycles of 94°C for 30 s, 55°C for 30 s, and 72°C for 1 min, with a final extension at 72°C for 10 min. The cycle threshold (Ct) value for a positive sample was set at 35 cycles.

**TABLE 1:** Primers and probes used for the identification of two novel viruses in this study

Virus	Type	Primer/probe	Sequence (5'to3')
Shandongifla-like virus 1	qRT-PCR	F	CTAAAGCCGCAGTTGAGGATAAAG
		R	TCTGACTACAATGAATGCACCCTT
		P	TCTCGACGACGCCATAATGCCTCTTC
Shandongifla-like virus 2	qRT-PCR	F	GCTGTTTAGTTTGAGTCTACCCTACGAT
		R	CTCACCAGCTATTGCGCCATTCCG
		P	GGTATCTATGTGTCGTCAACTCAACAC

### Data analysis and accession number

We compared the distribution of virus species richness between Culex and non-Culex mosquitoes using the Mann-Whitney U test (α = 0.05). Differences in virus carriage density at the individual level were assessed via permutation tests (10,000 replicates). Viral community diversity across host taxa was quantified using Simpson’s 1-D index (1 - ∑i = 1S pi^2^, where S = number of virus species and pi = proportion of the i^th^ virus in the host population). Permutation tests (10,000 iterations) evaluated differences in virus species per individual between urban and farm sites, whereas PERMANOVA (Bray-Curtis distance, 999 permutations) tested for compositional differences in viral carriage communities between these habitats (vegan package, R). Host-virus-environment interactions were visualized using chord diagrams (circlize package), with transmission networks reconstructed using directed arrows between two-colored environmental nodes. All analyses were conducted in R 4.3.1, with significance thresholds at *P* < 0.05. The predicted protein structures generated by AlphaFold were analyzed and annotated using PyMOL molecular visualization software. Virus prevalence was calculated using the minimum infection rate (MIR) formula: MIR = (number of positive pools/total number of mosquitoes tested) ×100. Statistical comparisons of positive rates were performed using Fisher’s exact test in GraphPad Prism (v5.00), with *P* values < 0.05 considered statistically significant.

The complete genome sequences of all novel viruses identified in this study have been deposited in GenBank under accession numbers PQ753142-PQ753143, PQ767078-PQ767080, PQ767084-PQ767085, and PQ901899-PQ901904.

## RESULTS

### Virome profiles of mosquitoes collected from Shandong

**Fig 1:**
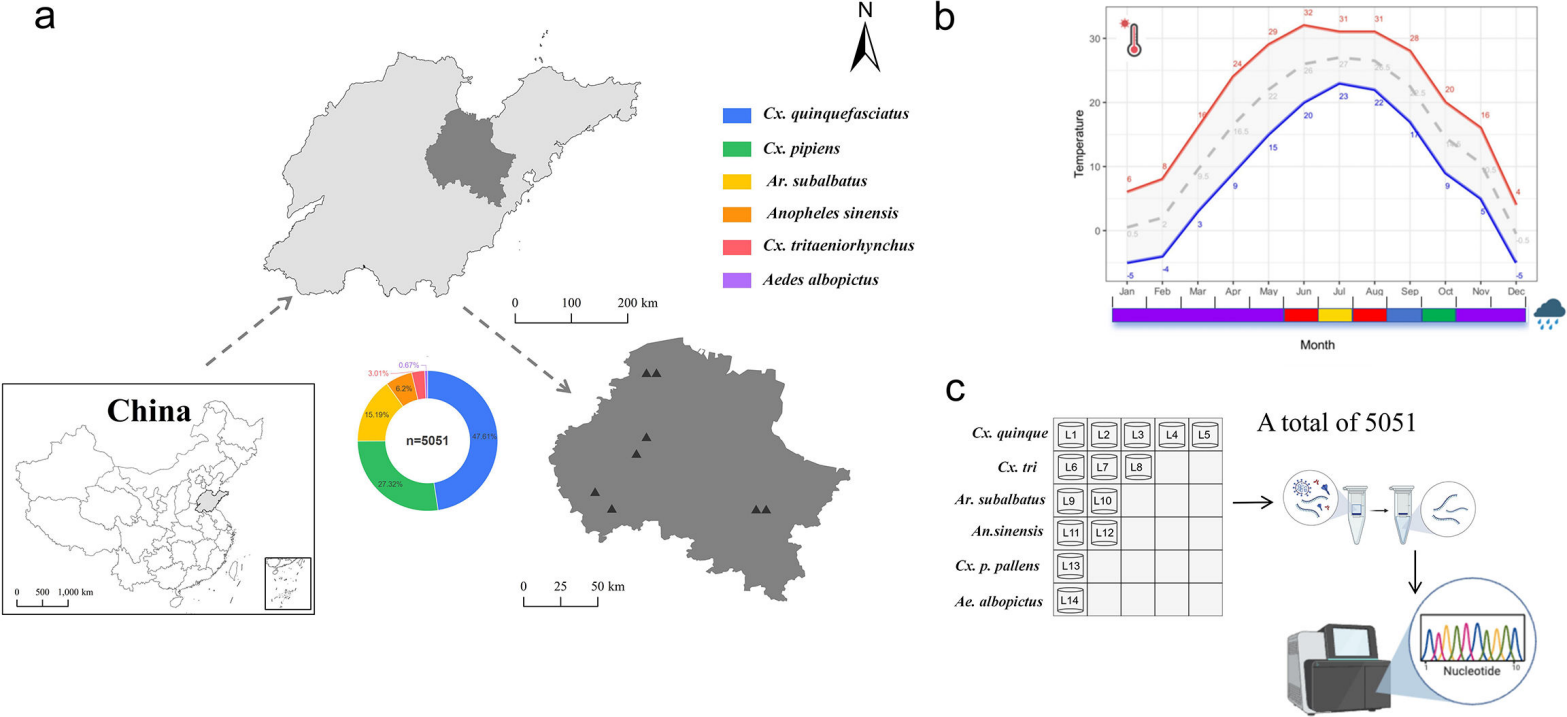
The map illustrates the geographical distribution of sampling points and the composition of mosquito species collected in Shandong Province, China. The color scheme represents the various mosquito species: blue for *Culex* leucocephalus, green for *Culex* albopictus, yellow for Aedes vexans, orange for *Anopheles sinensis*, red for *Culex tritaeniorhynchus*, and purple for *Aedes albopictus*. (**a**) Average weather conditions in Weifang. As reported by the China Weather website (https://www.tianqi24.com/weifang/history.html), the red line denotes the maximum temperature, the blue line represents the minimum temperature, and the dotted line indicates the monthly average temperature. The color-coded months signify different ranges of average monthly precipitation: purple (0–50 mm), green (50–100 mm), blue (100–150 mm), and red (>150 mm). (**b**) Workflow for processing the collected mosquito specimens. (**c**) Library construction and sequencing.

From June to August 2021, a total of 5,051 mosquitoes were collected from eight sampling sites in Weifang City, Shandong Province (Fig. 1a and b). Through morphological identification, these mosquitoes were classified into four genera and six species: *Culex quinquefasciatus*, *Culex pipiens pallens, Armigeres subalbatus, Anopheles sinensis*, *Culex tritaeniorhynchus*, and *Aedes albopictus*. To ensure taxonomic accuracy, a subset of representative specimens from each morphologically identified mosquito species was subjected to molecular confirmation. The COI gene was amplified by PCR and sequenced, yielding a 603 bp fragment for each sample. Sequence analysis was performed using BLASTn against the NCBI nucleotide database, with species identity confirmed when query sequences exhibited >98% homology to reference sequences of corresponding species. This molecular validation approach demonstrated complete concordance with our morphological identifications, reinforcing the reliability of our species classification. Among them, *Culex quinquefasciatus* accounted for 2,405 mosquitoes or 47.61% of the total number of mosquitoes captured, whereas *Culex trituberculatus* and *Aedes albopictus* accounted for a smaller number of 152 and 34 mosquitoes, representing 3.01% and 0.67% of the total number of mosquitoes captured, respectively. Depending on the collection sites and mosquito species, all mosquito samples were categorized into 105 pools, which were then constructed into 14 libraries following nucleic acid extraction (Fig. 1c; Table 2). High-throughput sequencing generated 105 GB of data for the 14 libraries. After removal of host sequences, 3,705,841,260 clean reads were obtained for downstream analyses, of which 35.3 million reads, or 0.95% of the total, were of viral origin. The remaining reads primarily originated from non-viral components in the samples, including environmental microbes (such as bacteria and fungi), mosquito symbionts (such as Wolbachia), and low-abundance host transcripts. Additionally, some unannotated sequences may have resulted from technical noise during the sequencing process (such as adapter remnants or low-complexity sequences). Eventually, a total of 9,612 viral contigs were obtained by *de novo* assembly and annotated to ten virus species belonging to eight families: *Birnaviridae, Botourmiaviridae, Iflaviridae, Narnaviridae, Picornaviridae, Peribunyaviridae, Secoviridae,* and *Tombusviridae*. No viruses were found integrated into the host genome, thus ruling out the presence of these viruses as endogenous viral elements (EVEs).

**TABLE 2:** qRT-PCR to determine the two novel viruses in mosquito samples of Weifang, Shandong Province, in 2021

Species	No. of mosquitoes	Site	No. of pools	Library	Shandong ifla-like virus 1		Shandong ifla-like virus 2		*P*
					No. of positive pools	MIR %	No. of positive pools	MIR %	
*Culex quinquefasciatus*	481	Urban area	10	L1	0	0	0	0	>0.05
	1924	Animal farm	39	L2- L5	15	0.62	6	0.25	
*Culex pipiens pallens*	828	Urban area	17	L6- L7	0	0	0	0	
	552	Animal farm	11	L8	4	0.29	2	0.14	
*Armigeres subalbatus*	767	Animal farm	16	L9 L10	0	0	0	0	
*Anopheles sinensis*	313	Animal farm	7	L11 L12	0	0	0	0	
*Culex tritaeniorhynchus*	152	Urban area	4	L13	0	0	0	0	
*Aedes albopictus*	34	Urban area	1	L14	0	0	0	0	
Total	5051		105	14	19	0.38	8	0.16	

### Diversity, genome, and phylogenetic analysis of mosquito viromes

**Fig 2:**
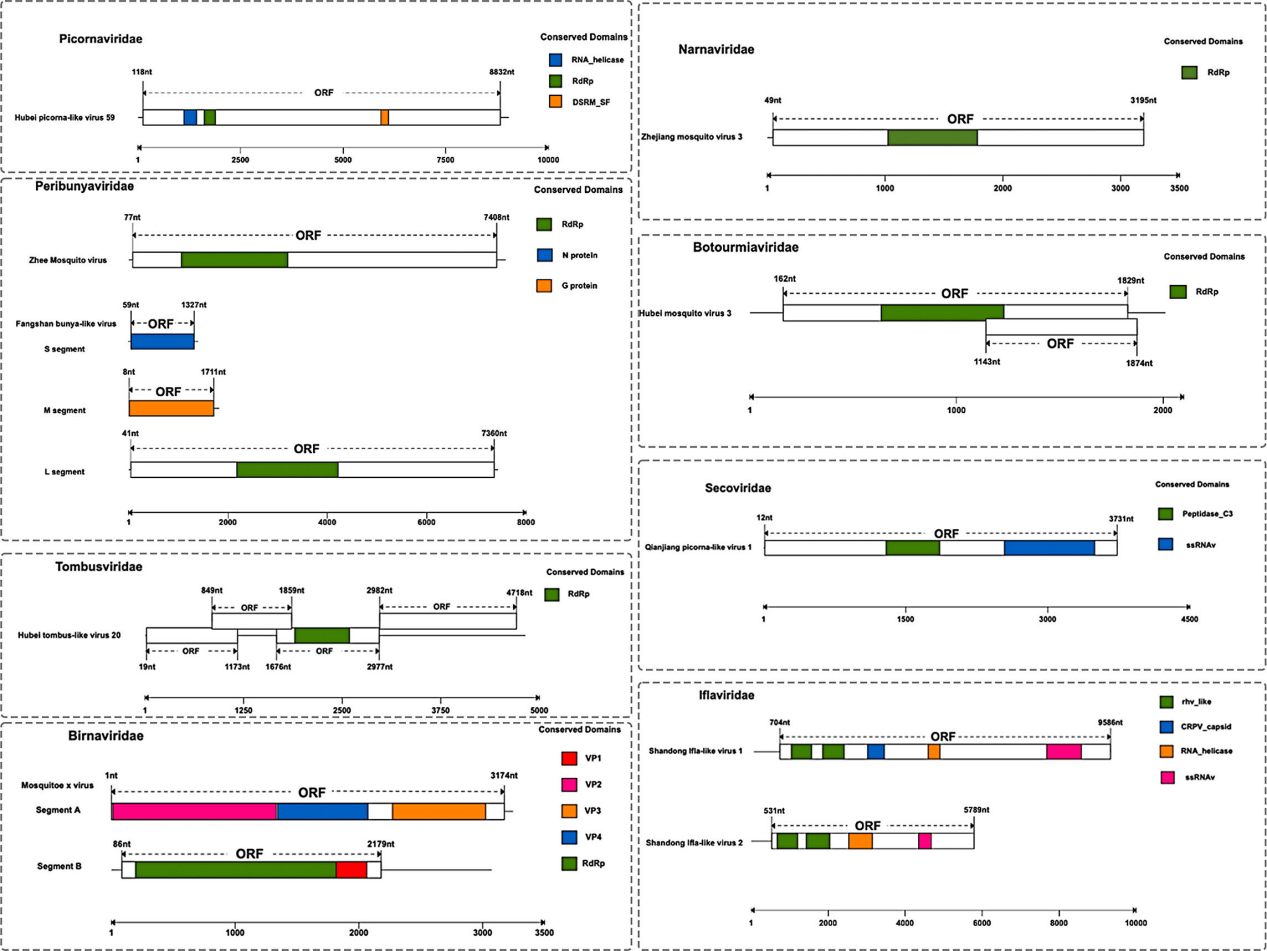
Schematic representation of the genome structures of ten newly discovered viruses belonging to eight distinct families, as revealed by metagenomic next-generation sequencing analysis of mosquito samples collected in Shandong Province, China.

**Fig 3:**
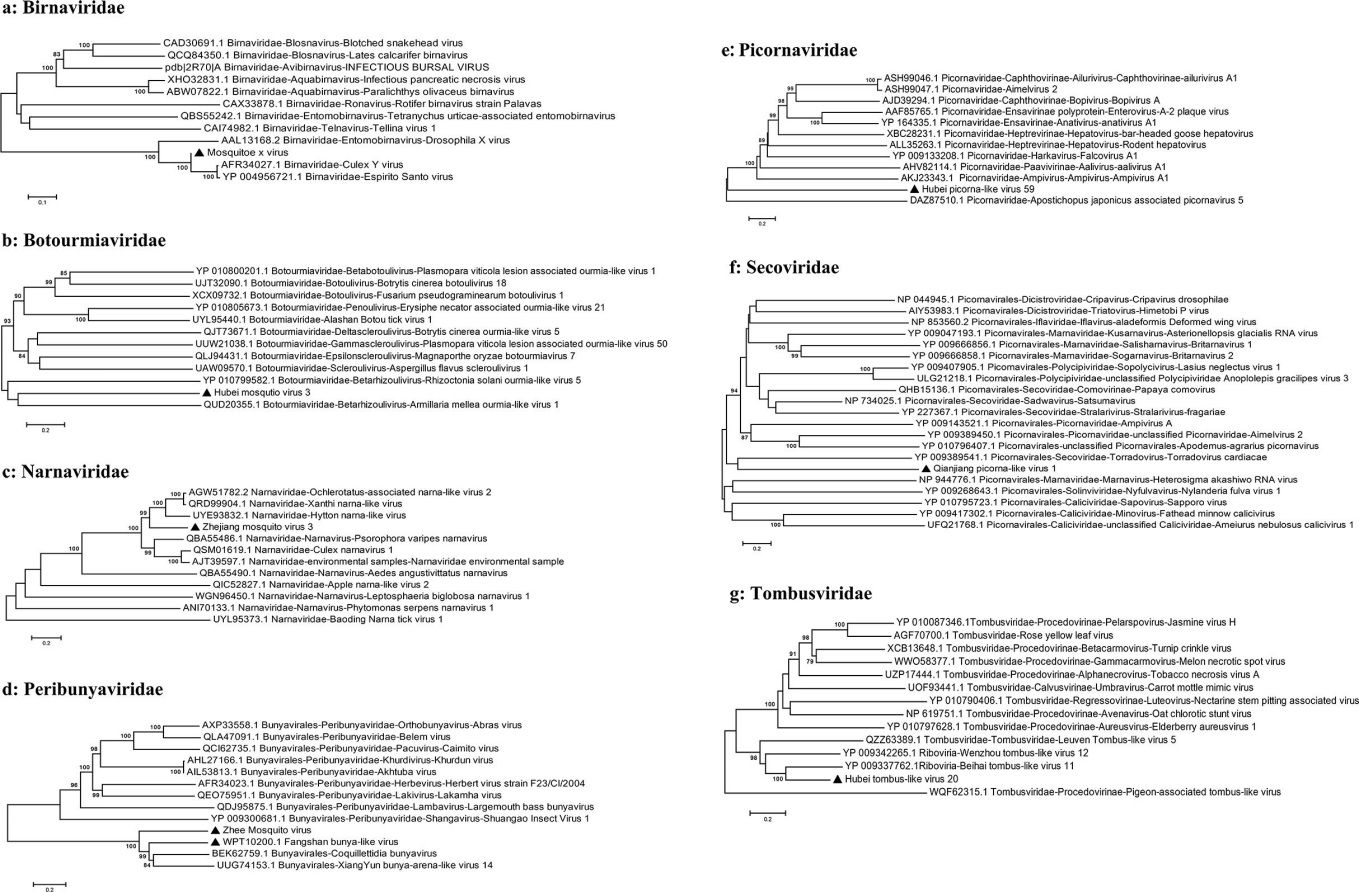
The figure consists of seven panels (**a-g**), each presenting a phylogenetic analysis of the viral sequences identified from mosquito samples collected in this study. Each panel illustrates the evolutionary relationships among various virus families using a phylogenetic tree. Viral sequences identified from the mosquito samples in this study are denoted by black triangles. The phylogenetic trees were constructed using the neighbor-joining (NJ) method implemented in MEGA 5.1, with 1,000 bootstrap replications. Bootstrap values exceeding 70% were regarded as statistically significant. The numbers displayed above the branches represent the bootstrap support values.

**Fig 4:**
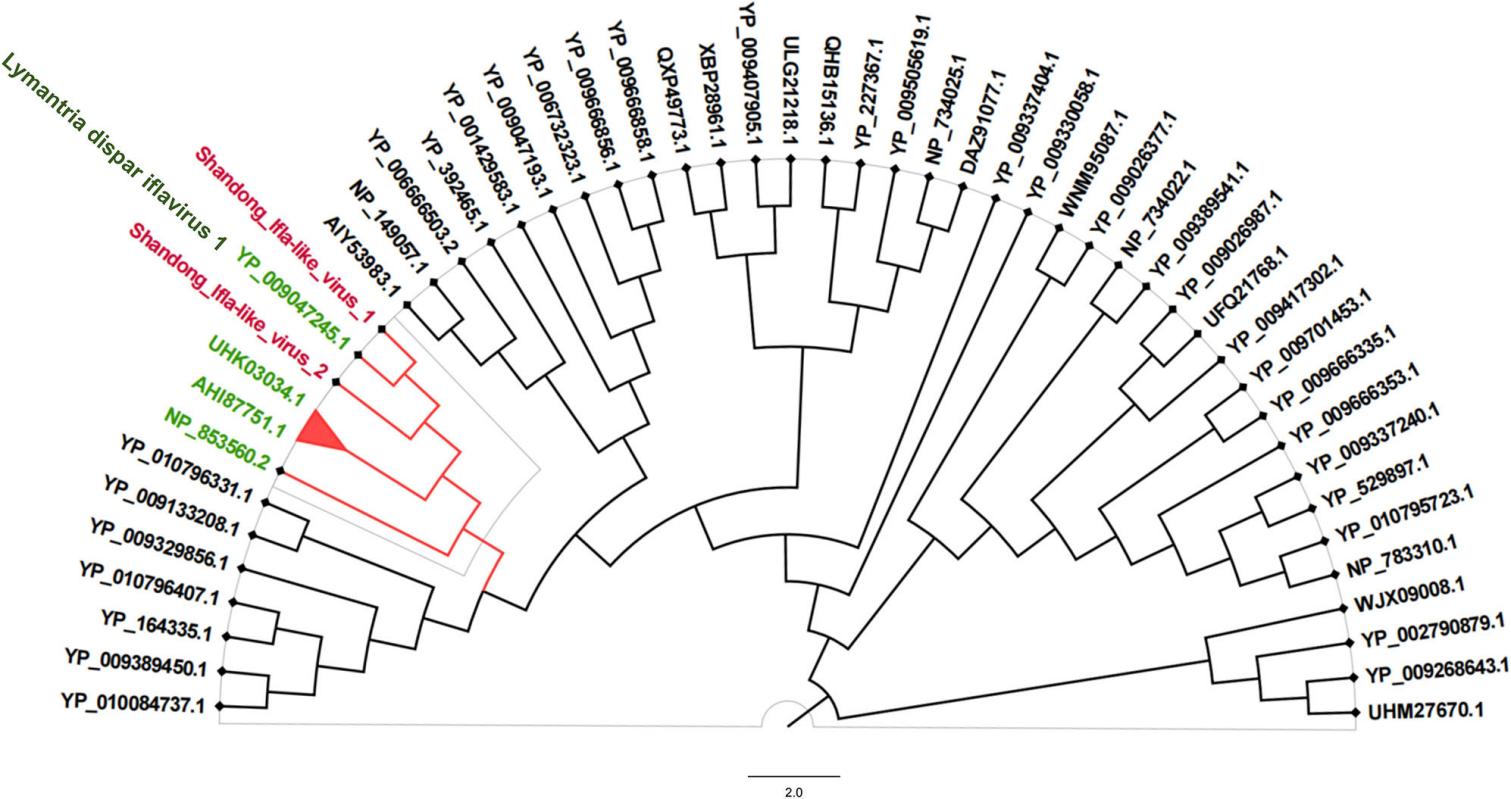
A phylogenetic tree was constructed based on viruses belonging to the order *Picornavirales* using the maximum likelihood method. The newly identified Shandong Ifla-like viruses and their close relatives are highlighted with red branches. Outgroup sequences from Lymantria dispar *Iflavirus* 1 are indicated by green labels. The scale bar represents 2.0 nucleotide substitutions per site. Accession numbers for all virus isolate sequences are provided.

**Fig 5:**
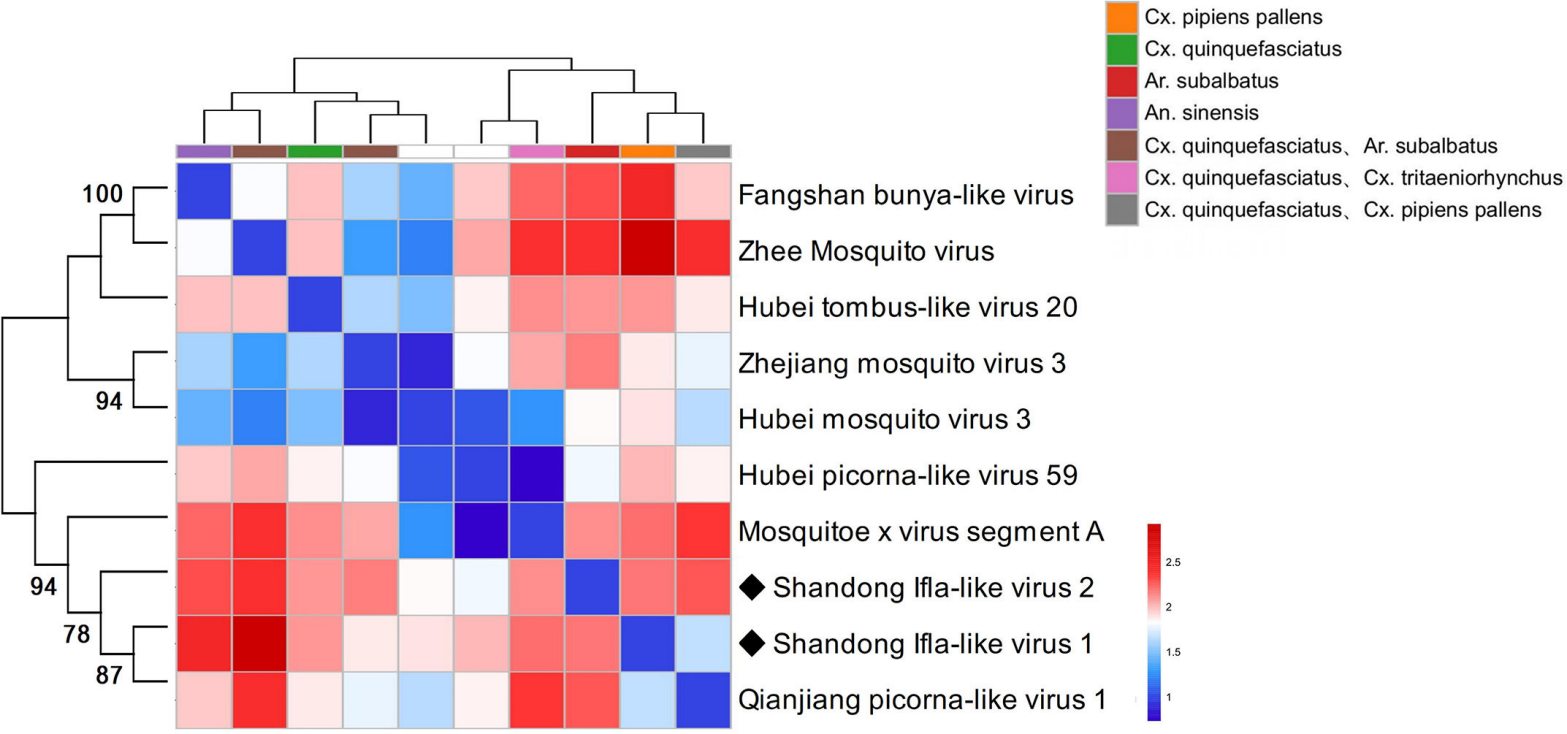
Cluster analysis and relative abundance of viral sequences isolated from mosquito pools. The pheatmap R package was employed to generate heatmaps visualizing correlation matrices of viral sequences across distinct mosquito populations. The left dendrogram represents the hierarchical clustering of viral sequences based on their evolutionary relationships, with the associated bootstrap values displayed at each node. The top dendrogram depicts the hierarchical clustering of mosquito populations based on their correlation. The colored bars at the top of the heatmap indicate the mosquito species corresponding to each sample. The color scale of the heatmap ranges from blue (indicating low similarity) to red (indicating high similarity), representing the degree of sequence similarity between the viral sequences.

In this study, we identified 10 viruses from mosquito samples, most of which had complete coding sequences (CDS) (Fig. 2). Among these, two viruses exhibited significant differences in their amino acid sequences compared to previously discovered viruses and were thus considered novel virus species, designated as Shandong Ifla-like virus 1 and Shandong Ifla-like virus 2. The remaining eight viruses, namely Hubei picorna-like virus 59, Zhee Mosquito virus, Hubei tombus-like virus 20, Mosquito x virus, Zhejiang mosquito virus 3, Hubei mosquito virus 3, Fangshan bunya-like virus, and Qianjiang picorna-like virus 1, have been identified and described in previous studies but have not yet been formally classified or approved by the International Committee on Taxonomy of Viruses (ICTV). This means that although these viruses have been detected and their sequences have been reported, they have not been officially reviewed, validated, or assigned a formal taxonomic status by the ICTV. These viruses were assigned to eight distinct families: *Birnaviridae*, *Botourmiaviridae*, *Iflaviridae*, *Narnaviridae*, *Picornaviridae*, *Peribunyaviridae*, *Secoviridae*, and *Tombusviridae*. Specifically, in *Birnaviridae* (14), we identified Mosquito X virus, a double-stranded RNA virus with a genome composed of two segments (A and B) encoding polyproteins. It clustered with Culex Y virus and Espirito Santo virus and is closely related to Punta Bolivar virus (Fig. 3a). In *Botourmiaviridae*, Hubei mosquito virus 3 was identified, with a single-stranded positive-sense RNA genome of 2,011 nucleotides (nt) containing two open reading frames (ORFs) encoding an RNA-dependent RNA polymerase (RdRp) and a hypothetical protein. It is closely related to Armillaria mellea ourmia-like virus 1 (Fig. 3b). In *Iflaviridae*, two novel viruses were identified: Shandong Ifla-like virus 1 and Shandong Ifla-like virus 2. Their genomes are single-stranded positive-sense RNA molecules, with lengths of 9,598 nt and 5,790 nt, respectively, encoding multiple functional proteins, including RNA helicase and CRPV capsid. They are closely related to Lymantria dispar Iflavirus 1 and form a monophyletic clade within the genus Iflavirus (Fig. 4). For *Narnaviridae*, Zhejiang mosquito virus 3 was identified (15, 16) with a genome of 3,226 nt containing two ORFs encoding an RNA-dependent RNA polymerase and a polyketide synthase domain protein. It clustered with Hyiton nama-like virus and Xanthi narna-like virus and is closely related to Psorophora varipes namavirus (Fig. 3c). In *Peribunyaviridae*, Fangshan bunya-like virus and Zhee Mosquito virus were identified. Their genomes consist of three segments (L, M, and S) encoding an RNA-dependent RNA polymerase, glycoprotein, and nucleocapsid protein, respectively. They are closely related to Shuangao Insect Virus 1 and form a monophyletic group with Coquillettidia bunyavirus (Fig. 3d). For *Picornaviridae*, Hubei picorna-like virus 59 was identified, with a single-stranded positive-sense RNA genome of 9,035 nt containing one ORF encoding a polyprotein. Phylogenetic analysis showed it is closely related to Ampivirus A1 (Fig. 3e). For *Secoviridae*, Qianjiang picorna-like virus 1 was identified, with a single-stranded positive-sense RNA genome of 4,125 nt containing one ORF encoding a polyprotein. It formed a monophyletic clade with Torradovirus cardiacae (Fig. 3f). Finally, for *Tombusviridae*, Hubei tombus-like virus 20 was identified, with a single-stranded positive-sense RNA genome of 4,832 nt containing four ORFs encoding hypothetical proteins and an RNA-dependent RNA polymerase. It is closely related to Beihai tombus-like virus 11 and Wenzhou tombus-like virus 12 and forms a clade with Leuven tombus-like virus 5 (Fig. 3g). Two-dimensional cluster analysis revealed specific virus-vector pairing preferences, with main clusters including the Pan Bunya-like virus clade (Fangshan bunya-like virus and Zhee Mosquito virus), the Hubei-Zhejiang virus complex, and the Shandong Ifla-like virus clustering group (Fig. 5). Notably, Hubei picorna-like virus 59 formed a distinct lineage, whereas Hubei tombus-like virus 20 clustered with the Hubei-Zhejiang virus complex, suggesting potential genetic recombination or convergent evolution across families. These viruses were predominantly detected in Culex mosquitoes, particularly in Culex quinquefasciatus and Culex pipiens pallens, indicating a distinctive host-virus coevolutionary relationship. These findings collectively enhance our understanding of the genetic diversity and evolutionary relationships within these virus families, providing valuable insights for future virological research and potential ecological studies.

### Coexistence of viruses in different mosquito species

**Fig 6:**
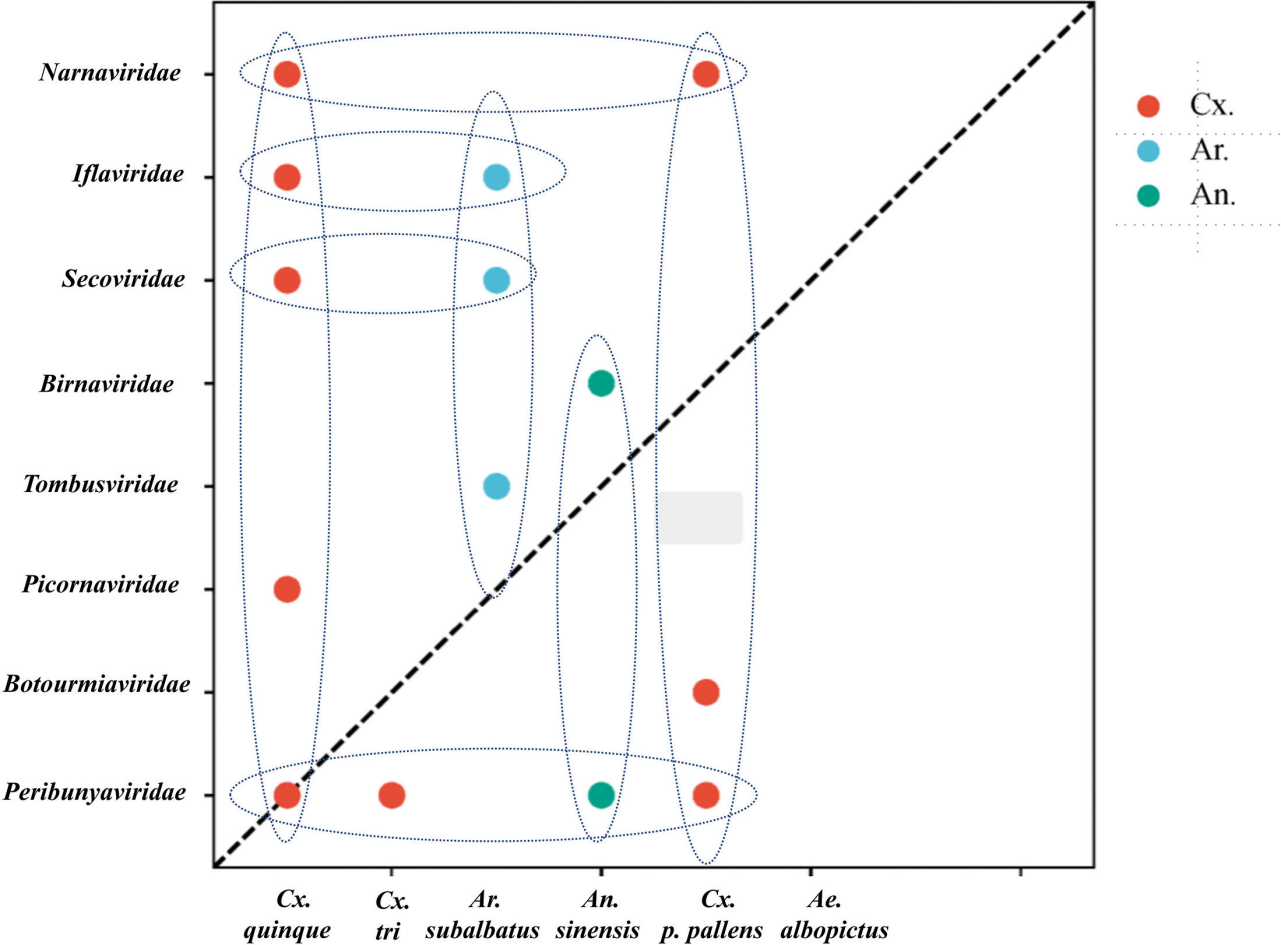
The graph depicts the relationships among various virus families investigated in this study. The x-axis represents the mosquito species, whereas the y-axis denotes the corresponding virus family names. Colored dots, labeled Cx., Ar., and An., symbolize the different host types associated with each virus species. Dashed ellipses illustrate the ecological associations between mosquito vectors and virus families.

The present study revealed a varying distribution of virus species among different mosquito species. Among the mosquito species examined, *Culex quinquefasciatus* harbored the highest diversity of viruses, with six distinct virus species identified. *Armigeres subalbatus*, *Anopheles sinensis*, *Culex pipiens pallens*, and *Culex tritaeniorhynchus* were found to carry four, two, two, and one virus species, respectively. In contrast, no viruses were detected in *Aedes albopictus* (Fig. 6). Analysis of the five *Culex quinquefasciatus* libraries (L1–L5) revealed a substantial proportion of reads originating from various virus families. The most prevalent virus families included *Picornaviridae* (35.2% of viral reads), *Narnaviridae* (7.4% of viral reads), *Peribunyaviridae* (16.8% of viral reads), *Iflaviridae* (18.1% of viral reads), and *Secoviridae* (3.7% of viral reads). These percentages represent the relative abundance of viral reads from the most prevalent families and do not account for all viral reads, as other virus families may also be present in smaller quantities. Further species-level analyses revealed that the majority of these viral reads were attributed to Hubei picorna-like virus 59, Zhejiang mosquito virus 3, Fangshan bunya-like virus, Shandong Ifla-like virus 1, Shandong Ifla-like virus 2, and Qianjiang Ifla-like virus 2. In three *Culex pipiens pallens* libraries (L6–L8), we identified the presence of Zhee Mosquito virus and Hubei mosquito virus 3. *Armigeres subalbatus* specimens also exhibited distinct viromes, predominantly comprising Hubei tombus-like virus 20, Shandong Ifla-like virus 1, Shandong Ifla-like virus 2, and Qianjiang picorna-like virus 1. Furthermore, we identified two Anopheles sinensis libraries (L11 and L12) and three *Culex pipiens pallens* libraries (L6–L8). Moreover, we identified Zhee Mosquito virus, Mosquito x virus, and Fangshan bunya-like virus in two Anopheles sinensis libraries (L11 and L12) and a Culex tritaeniorhynchus library (L13), respectively.

Furthermore, we observed the occurrence of identical virus species across various mosquito species. For instance, Zhee Mosquito virus was detected in both *Culex pipiens pallens* and *Anopheles sinensis* libraries; similarly, Zhejiang mosquito virus 3 was identified in *Culex quinquefasciatus* and *Culex pipiens pallens* libraries, and Fangshan bunya-like virus was found in *Culex quinquefasciatus* and *Culex tritaeniorhynchus* libraries. Shandong Ifla-like virus 1, Shandong Ifla-like virus 2, and Qianjiang picorna-like virus 1 were identified in the *Culex quinquefasciatus* and *Armigeres subalbatus* libraries, respectively.

### Background analysis of viral ecology

Host composition analysis revealed that mosquitoes of the genus *Culex* (*C. quinquefasciatus, C. pipiens pallens, and C. tritaeniorhynchus*) dominated the sampled population, collectively accounting for 78.0% (3,937/5,051) of all specimens, with *C. quinquefasciatus* representing the most abundant species (47.6%). Although *Culex* spp. harbored a greater diversity of virus species (8) compared with non-*Culex* spp. (4), statistical analysis using the Mann-Whitney U-test indicated no significant difference in virus species distribution at the species level (U = 7, *P* = 0.700). Similarly, an individual-based replacement test failed to detect significant variation (*P* = 0.312). Notably, Simpson’s diversity index suggested a more complex viral community structure in *Culex* mosquitoes (1-D = 0.781) relative to non-*Culex* species (1-D = 0.625). This observed trend may be attributed to the unique ecological traits of *Culex* spp. As a typical synanthropic mosquito genus, *Culex* preferentially breeds in organic-rich aquatic habitats, such as sewers and cisterns. The coexistence of various *Culex* species in these environments may facilitate the cross-species transmission of viruses by promoting interactions among pathogens from diverse host origins. Furthermore, the generalist feeding behavior of *Culex* spp.—which target birds, humans, and domestic animals, coupled with their high population density (78.0% of sampled individuals)—could amplify their role as viral "supercarriers" by increasing the frequency of host-vector contacts. Interestingly, the study did not detect *Aedes albopictus*, a container-breeding mosquito species. This absence may reflect its ecological specialization in small artificial water-holding containers, which likely limits its exposure to wildlife hosts and associated zoonotic viruses.

**Fig 7:**
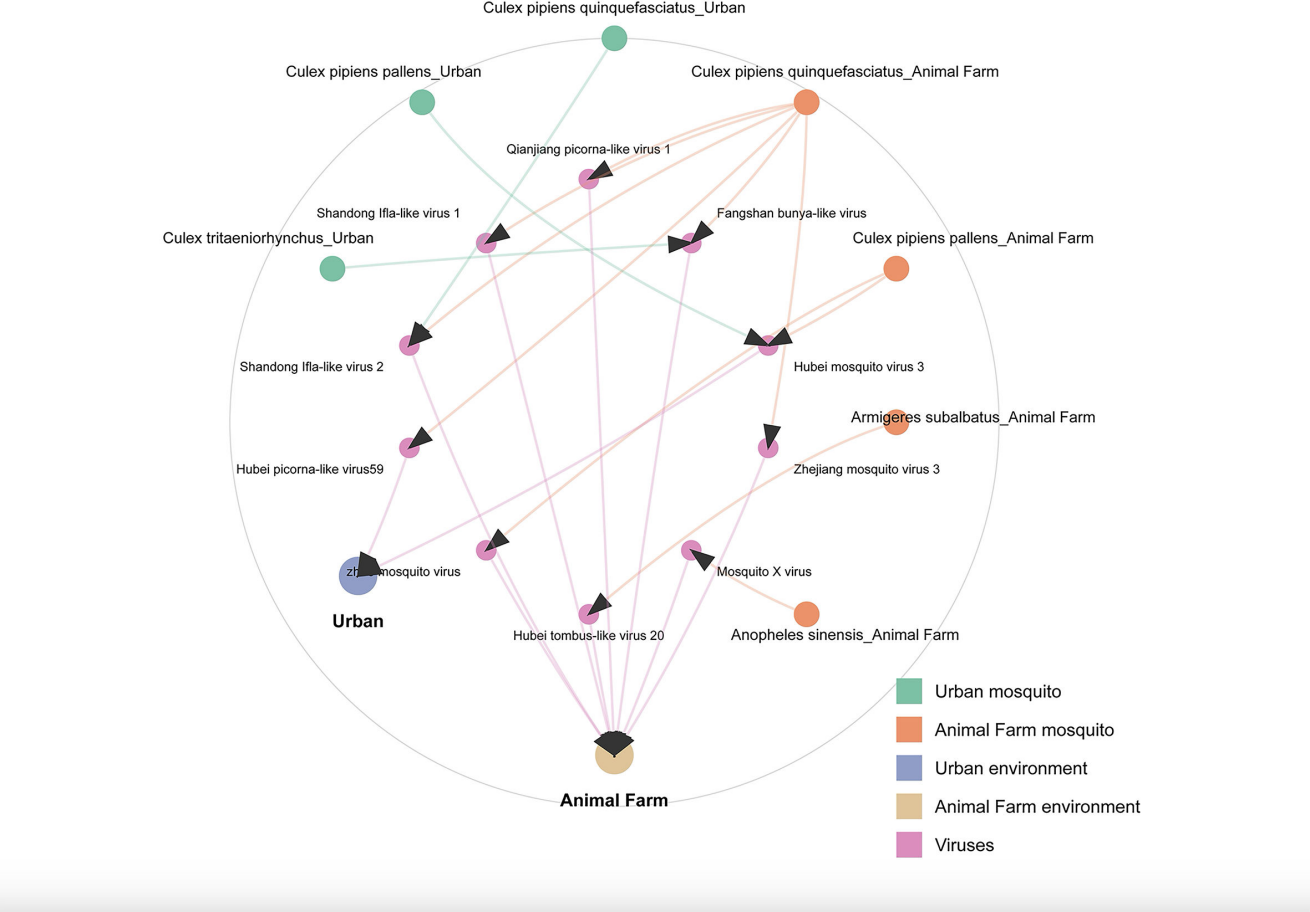
Host-virus-environment transmission network chord diagram. This chord diagram illustrates the ecological interaction network among mosquito hosts, virus species, and environmental contexts. Green and orange nodes represent mosquito species from distinct environmental habitats. Blue and pink nodes denote urban and farming environments, respectively, whereas purple nodes correspond to various viruses detected in the study. Arrows connecting the nodes indicate the direction of viral associations.

Comparative analysis revealed significant viral enrichment in farm environments compared with urban areas. Mosquitoes collected from farms carried a substantially higher virus species density (3.008 species/thousand individuals) than those from urban sites (0.656 species/thousand; permutation test, *P* < 0.001). Community structure analysis (PERMANOVA) further confirmed distinct viral carriage patterns between the two habitats (F = 24.0, *P* = 0.008). The observed disparity likely stems from multiple interacting ecological determinants: (i) intensive livestock farming operations serve as reservoirs for multi-host viruses, facilitating cross-species transmission; (ii) organic-rich fecal effluents establish optimal microhabitats that concurrently promote mosquito proliferation and viral persistence; and (iii) high-frequency interactions between dense host populations (e.g., livestock) and vector mosquito swarms create an amplification cascade that enhances viral diversity. Conversely, urban ecosystems demonstrate constrained viral diversity, attributable to both the paucity of wildlife reservoirs and the suppressive effects of anthropogenic chemical pollutants on aquatic breeding habitats (Fig. 7).

### Structural analysis of two novel viruses encoded proteins

**Fig 8:**
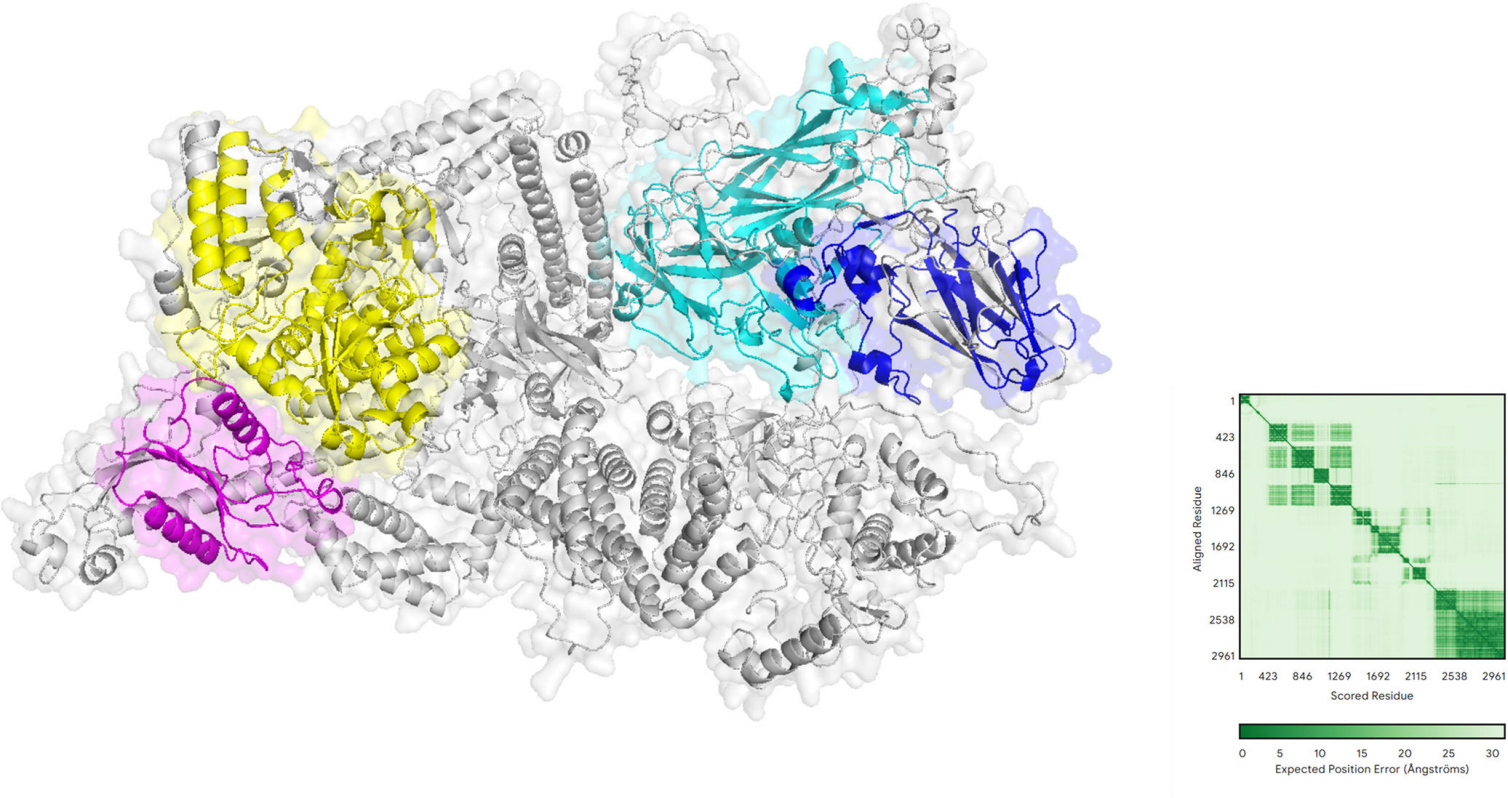
The predicted molecular structure of Shandong Ifla-like virus 1 is visualized, with various structural domains distinguished by different colors: yellow for *RdRp*, magenta for RNA-helicase protein, cyan for rhv-like protein, and blue for CRPV protein. The core structure is represented in gray. The predicted aligned error, shown on the right, illustrates sequence residue correlations using a green scale, where darker green shades signify stronger correlations. The coordinates suggest that the visualized data represent either protein interaction or structure mapping information.

We conducted comprehensive structural and functional analyses of the protein-coding regions of two novel viruses. AlphaFold prediction results revealed that Shandong Ifla-like virus 1 possesses a complex multi-structural domain architecture (Fig. 8). Among these domains, the *RdRp* structural domain displays a right-handed helical topology, a characteristic feature of viral polymerases, whereas the highly conserved A-F motif ensures optimal positioning for the catalytic reaction. Notably, the two adjacent Rhv-like structural domains exhibit a β-barrel conformation, indicating their potential involvement in genome encapsidation or host factor recruitment. The deconjugase structural domain adopts a RecA-like fold, potentially facilitating RNA deconjugation during genome replication. Interestingly, the compact globular structure of the CRPV structural domain implies its potential involvement in mediator-specific interactions, which are crucial determinants of arbovirus host range. Prediction alignment error analysis demonstrates that the model exhibits a remarkably high confidence (<10 Å) in the core catalytic region, especially within the *RdRp* structural domain. The figure illustrates a diverse array of inter-structural domain interactions, indicating that the enzyme activity may be subject to variable regulation.

**Fig 9:**
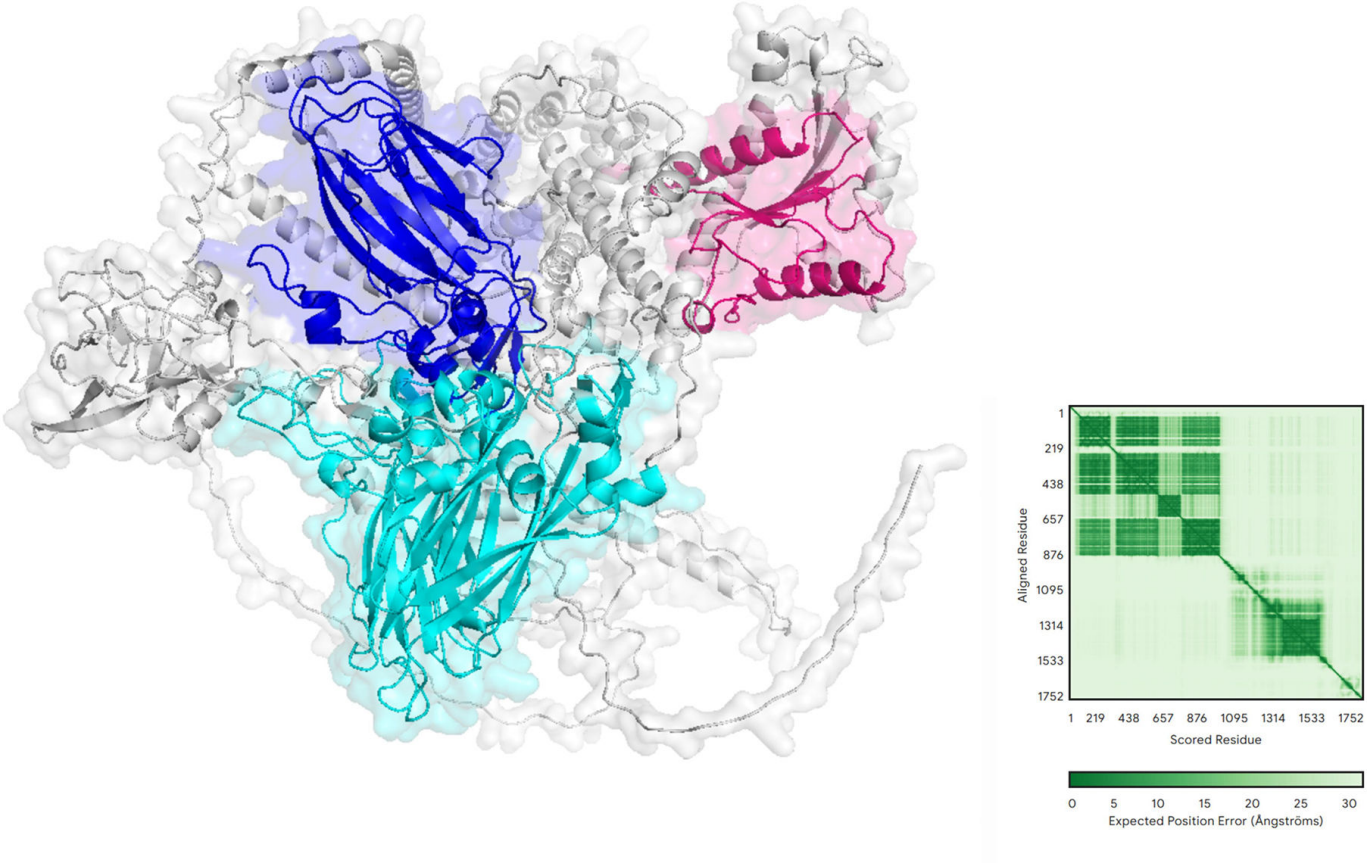
The predicted molecular structure of Shandong Ifla-like virus 2 is visualized, with various structural domains distinguished by different colors: magenta for RNA-helicase protein, cyan for rhv-like protein, and blue for CRPV protein. The core structure is represented in gray. The predicted aligned error, shown on the right, illustrates sequence residue correlations using a green scale, where darker green shades signify stronger correlations. The coordinates suggest that the visualized data represent either protein interaction or structure mapping information.

Concurrently, we identified four primary functional domains in Shandong Ifla-like virus 2 (Fig. 9). Among these domains, the dehelicase structural domain likely plays a crucial role in viral genome replication through ATP-dependent nucleic acid deconvolution. This activity is indispensable for viral RNA processing throughout the infection cycle. The CRPV_capsid and rhv-like structural domains are vesicle-associated components that are potentially involved in the assembly and structural integrity of viral particles. Prediction error analysis elucidates the distribution of protein structure prediction confidence. The rhv protein and CRPV regions exhibited low PAE values (<10 Å) and substantial cross-interacting regions, indicating a potentially stable structural coupling. Despite the accurate prediction of the internal conformation of the Helicase structural domain, the higher PAE values observed with other structural domains suggest its potential involvement in the regulation of viral function through dynamic conformational changes.

### Detection of the genomes of two novel viruses in mosquito samples

We screened all 105 mosquito pools for the presence of nucleic acids from these two viruses. QRT-PCR results revealed that 19 and 8 pools tested positive for Shandong ifla-like virus 1 and Shandong ifla-like virus 2, respectively. Both viruses were found to be associated with two mosquito species, namely *Culex quinquefasciatus* and *Armigeres subalbatus*. The minimum infection rates (MIRs) are calculated using the formula: MIR = (Number of positive pools/Total number of mosquitoes tested) × 100. The positivity rates are calculated using the formula: Positivity Rate = (Number of positive pools/Total number of pools) × 100. For Culex quinquefasciatus, the MIR for Shandong ifla-like virus 1 is 0.62% (15/2,405), and for Shandong ifla-like virus 2, it is 0.25% (6/2,405). For Armigeres subalbatus, the MIR for Shandong ifla-like virus 1 is 0.29% (4/1,380), and for Shandong ifla-like virus 2, it is 0.14% (2/1,380). The positivity rates are determined based on the number of positive pools out of the total pools tested for each species. No statistically significant differences were observed in the positivity rates of the two viral nucleic acids between the mosquito species.

## DISCUSSION

Mosquito-borne viral infectious diseases, characterized by their natural pathogenicity, are predominantly zoonotic in nature. Predicting the epidemic trends of these diseases is challenging, and they pose a severe threat to public health and safety (17, 18). The most common pathogenic mosquito-borne viruses include dengue virus (DENV), Zika virus (ZIKV), yellow fever virus (YFV), West Nile virus (WNV), and Japanese encephalitis virus (JEV), among others (19, 20). Furthermore, recent years have witnessed the continuous discovery of novel, unclassified viruses in mosquitoes (21). Consequently, our understanding of the mosquito virome remains incomplete. Virome surveillance significantly enhances our comprehension of the entire mosquito-associated virus pool by enabling the monitoring of known zoonotic pathogens and emerging viruses (15). Moreover, it has positive implications for our understanding of the ecological characteristics of viruses and their potential hazards to public health safety.

In this study, we performed species identification and genus-level analysis of the viromes of six common mosquito species in Weifang City, Shandong Province, China. We identified complete coding region sequences or RNA-dependent RNA polymerase (*RdRp*) sequences of 10 species, including two novel viruses that have not been previously reported. These viruses can be classified into eight virus families, demonstrating the extensive diversity of viruses harbored by mosquito vectors. This finding is consistent with numerous studies conducted in other regions worldwide. For instance, Binit Lamichhane et al. identified 42 virus species in 19 families from six mosquito species collected in Western Australia (22). Similarly, Maja Stanojević et al. discovered nine mosquito-associated viruses in seven families from mosquito species collected in Belgrade, Serbia (23). Collectively, these studies indicate that mosquitoes serve as reservoirs for a wide array of viruses, playing a crucial role in their replication and transmission life cycles. Furthermore, the viruses harbored by the same mosquito vectors within the same region exhibit variation. For example, Yuhao Wang’s 2021 study revealed that the eight virus families carried by four common mosquito species in the Shandong region had minimal overlap with those identified in the present study (24). This finding further highlights that the capacity and diversity of viral reservoirs harbored by mosquitoes are far greater than previously recognized. Regarding virus classification, the majority of viruses identified in this study are not pathogenic to humans or animals and likely belong to mosquito-specific viruses, which maintain a symbiotic relationship with their insect hosts. This finding is unsurprising, as numerous medically important mosquito-borne viruses replicate at low levels in mosquitoes, except during epidemic outbreaks when their prevalence and abundance may increase significantly. Furthermore, numerous studies have demonstrated that human and animal viral pathogens in mosquito vectors may constitute a minor fraction of the mosquito virome. Using metagenomic analysis, we identified two viruses, Fangshan bunya-like virus and Zhee Mosquito virus, which are closely related to the Shangavirus genus within the family Peribunyaviridae. Although these viruses are classified within the Shangavirus genus, it is important to note that this genus primarily contains insect-specific viruses and is not known to include any pathogens that are harmful to humans or animals. Therefore, although our findings expand the known diversity of viruses within this genus, they do not suggest a direct pathogenic risk to humans or animals (25). However, this hypothesis requires further confirmation through functional experiments.

Based on the sequence analysis results, we assembled the complete coding region sequences of these eight viruses and subsequently performed genomic analysis. Among these viruses, Zhee Mosquito virus is a single-stranded negative-sense RNA virus, Fangshan bunya-like virus is a three-segmented single-stranded negative-sense RNA virus, Mosquito X virus is a double-stranded RNA virus, and the remaining viruses are single-stranded positive-sense RNA viruses. These findings demonstrate that the majority of virus species harbored by mosquitoes are single-stranded RNA viruses. This observation may be attributed to the fact that most viruses harbored by mosquitoes are either plant viruses or non-pathogenic mosquito-specific viruses, which are predominantly positive-sense RNA viruses. Similar findings have been reported in other studies (26). However, with the exception of the Mosquito X virus, which has been classified at the genus level, the other seven known viruses have not undergone comprehensive viral taxonomic analysis. After performing conserved domain analysis on these eight viruses, we discovered that nearly all of them possess complete RNA-dependent RNA polymerase (*RdRp*) coding sequences. Consequently, we successfully constructed a phylogenetic tree based on the *RdRp* sequences. Utilizing the existing *RdRp* reference sequences of related viruses, we classified all eight viruses at the genus level. Given that *RdRp* is relatively conserved among viruses within the same family and possesses a high molecular weight, comparing *RdRp* homology is theoretically more reliable for determining the evolutionary relationships among viruses. The phylogenetic analyses revealed that these viruses are all novel members of a genus within their respective families, and these findings provide a foundation for future studies on the biological properties of each virus. Moreover, it is intriguing to note that we discovered the same virus can originate from entirely different species. For instance, Hubei picorna-like virus 59 and Hubei tombus-like virus 20 were initially discovered in spiders, whereas the present study identified them in *Culex quinquefasciatus* and *Armigeres subalbatus*, respectively. Similarly, Qianjiang picorna-like virus 1 was initially thought to originate from Procambarus clarkii, but we detected its presence in both *Culex quinquefasciatus* and *Armigeres subalbatus*. This phenomenon suggests that the host range of these three viruses is not restricted to a single species, and they exhibit a broad infectious cell spectrum. These viruses are capable of cross-host infection and transmission. However, the potential influence of mosquito-spider or mosquito-Procambarus clarkii food chain factors cannot be excluded.

In this study, we identified two novel viruses, which we provisionally named Shandong Ifla-like virus 1 and Shandong Ifla-like virus 2. Genomic analysis revealed that Shandong Ifla-like virus 1 is a single-stranded RNA virus possessing an open reading frame that encodes five proteins. However, further investigation is required to determine whether these proteins are expressed independently or through polyprotein cleavage. In contrast, the complete coding region sequence of Shandong Ifla-like virus 2 was not obtained; instead, only an incomplete ORF lacking the *RdRp*-related sequence information was identified. The genomic framework and conserved regions of Shandong Ifla-like virus 1 and Shandong Ifla-like virus 2 exhibit a high degree of similarity, indicating a potential genetic relationship between the two viruses. Sequence similarity analysis revealed that the amino acid sequence of the coding region of Shandong Ifla-like virus 1 shared the highest identity (82.67%) with Lymantria dispar *Iflavirus* 1. In contrast, the amino acid similarity between Shandong Ifla-like virus 2 and Xi'an Ifla-like virus was only 27.31%. These findings suggest that Shandong Ifla-like virus 1 and Shandong Ifla-like virus 2 are likely novel mosquito-associated viruses. Furthermore, phylogenetic analyses suggested that Shandong Ifla-like virus 1 and Shandong Ifla-like virus 2 likely represent novel members of the genus *Iflavirus* within the family *Iflaviridae*. The discovery of these viruses expands the known diversity of arboviruses and highlights the vast number of undiscovered virus species in nature. The minimum infection rates (MIR) of mosquitoes carrying Shandong Ifla-like virus 1 (0.38%) and Shandong Ifla-like virus 2 (0.16%) suggest that these viruses are present in the local mosquito populations, albeit at relatively low levels. This indicates that although the viruses have established a presence, they are not widely distributed among the mosquito populations. This finding highlights the need for continued monitoring to better understand the dynamics of these viruses in the region. This finding is consistent with our previous study on Huberti mosquito virus 2 (HMV2) in Shandong province (27). These results indicate that the viruses have adapted to the local environment and established a sustainable and stable viral life cycle. Furthermore, no statistically significant difference was observed in the infection rates of Shandong Ifla-like virus 1 and Shandong Ifla-like virus 2 among different mosquito species, suggesting that these viruses can infect and replicate in multiple mosquito species without exhibiting a clear host preference.

Numerous mosquito species are capable of harboring multiple viruses concurrently, demonstrating their role as efficient vectors for a wide range of viruses and their significance as a substantial viral reservoir. Although the majority of the viruses identified in this study are not arboviruses, the presence of diverse viral communities underscores the importance of mosquitoes as vectors for various pathogens. Our findings corroborate this notion; for instance, six virus species from five distinct families were identified in *Culex quinquefasciatus*, whereas *Armigeres subalbatus*, *Anopheles sinensis*, and *Culex pipiens pallens* were each found to harbor more than two types of viruses. This diversity may be closely linked to the ecological niches occupied by these mosquito species. Both male and female mosquitoes feed on plant nectar and fruit juices, whereas female mosquitoes additionally consume human or animal blood prior to oviposition. Consequently, mosquitoes acquire and transmit a diverse array of viruses from multiple sources through complex interactions. Interestingly, no viruses were detected in the *Aedes albopictus* mosquitoes examined in this study, which might be attributed to the limited sample size collected for this particular species. Furthermore, as multiple mosquitoes of the same species were pooled together, it was not feasible to conduct sequencing analysis on individual mosquitoes. Consequently, only the viral diversity within each mosquito species could be ascertained, whereas the presence of multiple viruses within a single mosquito could not be determined. Moreover, we have identified the presence of several viruses, such as Zhee Mosquito virus and Fangshan bunya-like virus, in two distinct mosquito species. This finding suggests that these viruses are not limited to a single vector species and can be transmitted by multiple mosquito species. Furthermore, the possibility that these viruses may be present in additional mosquito species cannot be excluded. These observations also imply that the viruses in question may have a broader geographical distribution and enhanced transmission potential.

This study has several limitations that should be acknowledged. First, due to the small genome sizes of certain viruses identified in this study, it is possible that we have incomplete genome sequences. These incomplete sequences may contain additional open reading frames (ORFs) that were not identified in our analysis. For example, the ORF of Shandong Ifla-like virus 2 is incomplete, which may limit our understanding of its full genomic structure and potential functions. Second, the pathogenic potential of the two novel viruses, Shandong Ifla-like virus 1 and Shandong Ifla-like virus 2, was not thoroughly investigated. Given that the Iflaviridae family primarily contains insect-specific viruses, it is more appropriate to focus future studies on understanding their impact on mosquito populations and their ecological significance, rather than their pathogenic potential in animal models. Third, we were unable to isolate or culture the virus from the samples. The obtained sequences likely represent either partial genomic fragments or dominant viral populations, which constrains our ability to investigate viral infectivity, tissue tropism, and pathogenic potential. Furthermore, this approach may have overlooked minority viral variants present at lower abundance. In the future, we plan to focus on acquiring live virus strains to enable more in-depth pathogenicity studies and to conduct vector competency studies to better understand the transmission potential of these viruses.

### Conclusions

In this study, we employed high-throughput sequencing techniques to comprehensively analyze the diversity and abundance of viruses present in mosquito populations from Weifang, Shandong Province, providing foundational data on mosquito-associated viruses in the region. The generated data not only expand our understanding of the diversity of mosquito-borne viruses but also contribute to the investigation of the relationships among mosquitoes, viruses, and environmental factors, ultimately deepening our knowledge of the mosquito infectome.

## Data Availability

All sequence data generated and analyzed in this study are available at the NCBI (https://www.ncbi.nlm.nih.gov).
